# *In‐situ* glial cell‐surface proteomics for normal brain aging

**DOI:** 10.1002/alz.087477

**Published:** 2025-01-03

**Authors:** Madeline P Burns

**Affiliations:** ^1^ Baylor College of Medicine, Houston, TX USA

## Abstract

**Background:**

During aging, we observe several changes in the brain that render it more vulnerable to a variety of age‐related neurodegenerative diseases, including Alzheimer’s Disease. Glia‐neuron interactions mediate brain development and physiology via cell‐surface proteins at the cell‐surface interface, and dysregulation of these interactions is considered one of the hallmarks of brain aging. Due to the critical role glial cells play in neuroplasticity, immune function, and homeostasis, dysregulation of glial cell‐surface proteins is hypothesized to contribute to neurodegeneration. However, it remains technically difficult to profile glial cell‐surface proteomes from intact brains, which is necessary to preserve important neuron‐glia global dynamics.

**Method:**

Recently, we employed a state‐of‐the‐art *in‐situ* cell‐surface proteomics technique to profile how glial cell‐surface proteomes change during normal brain aging in young and old flies.

**Result:**

In total, we identified 872 glial cell‐surface proteins, and found that while most up‐regulated genes were associated with cell localization and transport, most down‐regulated genes were associated with neural development, circuit organization, and neuroplasticity. Based on our proteomic profiles, we selected 48 candidate genes for further screening (based on which were most up‐ or down‐regulated). Currently, we are conducting several gain‐of‐function/loss‐of‐function genetic screens to investigate the effects of each candidate gene on lifespan, using the repo‐GS/UAS binary system, and have selected two candidate genes of interest to investigate further.

**Conclusion:**

We have found that some of the most differentially expressed genes were associated with neural development and wiring molecules not previously thought to be particularly active in glia.

